# Impact of micro/nano cadmium oxide on shielding properties of cement–ball clay matrix

**DOI:** 10.1038/s41598-023-45516-2

**Published:** 2023-10-25

**Authors:** Mona M. Gouda, Mahmoud I. Abbas, Malak H. Eid, Mohamed S. Ziedan, Moaaz A. Ibrahim, Mohamed M. Tawfik, Ahmed M. El-Khatib

**Affiliations:** Physics Department, Faculty of Science, Alexandria University, Alexandria, 21511 Egypt

**Keywords:** Nanoscience and technology, Physics

## Abstract

This study investigates the gamma radiation shielding properties of cement–ball clay matrix composites doped with micro- and nano-sized cadmium oxide (CdO) particles. The linear attenuation coefficient (LAC) was determined using a sodium iodide (NaI) detector and five radioactive point sources with energies ranging from 59.5 to 1408 keV. The LAC values obtained were compared to the XCOM database and found to be in good agreement. The composites' half-value layer (HVL), tenth value layer (TVL), mean free path (MFP), effective atomic number (Z_eff_), equivalent atomic number (Z_eq_), and absorption buildup factor (EABF) were determined. The results showed that the addition of CdO particles improved the radiation-shielding behavior of the composites and increasing the weight fraction of CdO particles increased the shielding effectiveness. The results also illustrated that when nano-sized CdO particles were compared to their micro-sized counterparts, there was a significant enhancement in radiation shielding effectiveness. For instance, a composite material composed of 50% cement, 41.7% ball clay, and 3.8% nano CdO at an energy level of 0.0595 MeV exhibited a remarkable 12.2% increase in attenuation, surpassing the performance of the micro-sized sample with an equivalent concentration. Similarly, another composite consisting of 50% cement, 33.3% ball clay, and 16.7% nano CdO demonstrated a significant 15.4% increase in attenuation at the same energy level, when compared to the micro-sized sample. The study demonstrates the potential of CdO-doped cement–ball clay matrix composites for gamma radiation shielding applications.

## Introduction

A plurality of nations throughout the world consider nuclear technology a replacement for fossil fuels. Studying the ability of some commonly accessible building materials, including concrete, rocks, and clay, to absorb gamma rays is required due to the expanding usage of radioisotopes in many medical and industrial domains^1–4^.

Since ancient times, clay has been utilized all over the world to build civilizations that are of good quality and weather-tolerant; furthermore, clay has refractory properties such as a high melting point, thermochemical stability, and thermal shock resistance^5^. When compared to cement, clay items like ceramic pots, fired bricks, and tiles are more affordable, longer lasting, and environmentally safe construction materials. That made them widely accessible at cheap rates in many regions of the world.

High atomic numbers and high-density materials are widely proven to be effective ionizing shields. Lead is the most often applied material for these uses^6–9^. As clay is non-toxic, it is suitable as a radiation shielding material; on the contrary, lead is a toxic element, chemically unstable, and heavy^10^. Therefore research on new materials that are less toxic and cost-effective, besides preserving the environment, is required to protect against radiation^11^.

In recent years, nanoparticles have had very small intermolecular distances between molecules, so the possibility of photon collisions with the material's atoms increases, which improves the material's ability to attenuate photons as a result. Due to their promising qualities, like their lightweight nature and desirable mechanical, chemical, and physical properties^12,13^, the application of nanomaterials in numerous branches of technology and science has caught the attention of scientists. Researchers have been actively investigating the incorporation of nanoparticles as fillers in building materials as cement and clay matrices and other ones; aiming to enhance the protective properties of these building materials. In the realm of nuclear engineering, there is a significant interest in nanocomposites containing metals or heavy element oxides^14–16^. The conclusion is that CdO nanoparticles have better gamma radiation shielding abilities than micro-CdO particles. Their research has therefore concentrated on creating these nanocomposites as an alternative to conventional radiation shielding^17^. Almost all earlier studies concentrated on creating specific types of clay with certain heavy oxides for use as radiation shields^18^, while relatively few studies concentrated on creating clays with certain nano-scale heavy oxide particles^19,20^.

The authors of this study aimed to explore the radioprotective properties of ball clay from the Aswan region of Egypt. Ball clay is a clean and environmentally friendly building material that can be utilized in radiation protection applications. It can also be added to concrete mixtures as a substitute for sand, increasing its density and enhancing gamma-ray attenuation. The high melting point of this clay suggests its potential thermal stability when exposed to high-energy radiation, and its compressive strength makes it suitable for producing robust shielding materials. Given the growing demand for investigating the impact of filler size on gamma radiation shielding properties in various composite systems, further research is necessary^21–25^.

Knowing that cement and ball clay are widely used in construction, but understanding their behavior when mixed with CdO is a whole other thing. Therefore, the primary objective of this study is to study the mechanical properties of composites. Also, to examine the influence of particle size and weight percentage of CdO particles on the gamma radiation shielding capability of CdO/ball clay/cement composites. The radiation shielding parameters of different composites with different weight fractions were evaluated at photon energies ranging from 59.53 to 1408.01 keV using a NaI scintillation detector. Additionally, a comparative study was conducted to compare the radiation shielding abilities of nanoCdO and microCdO composites.

## Materials and methods

### Materials

**Figure 1:**
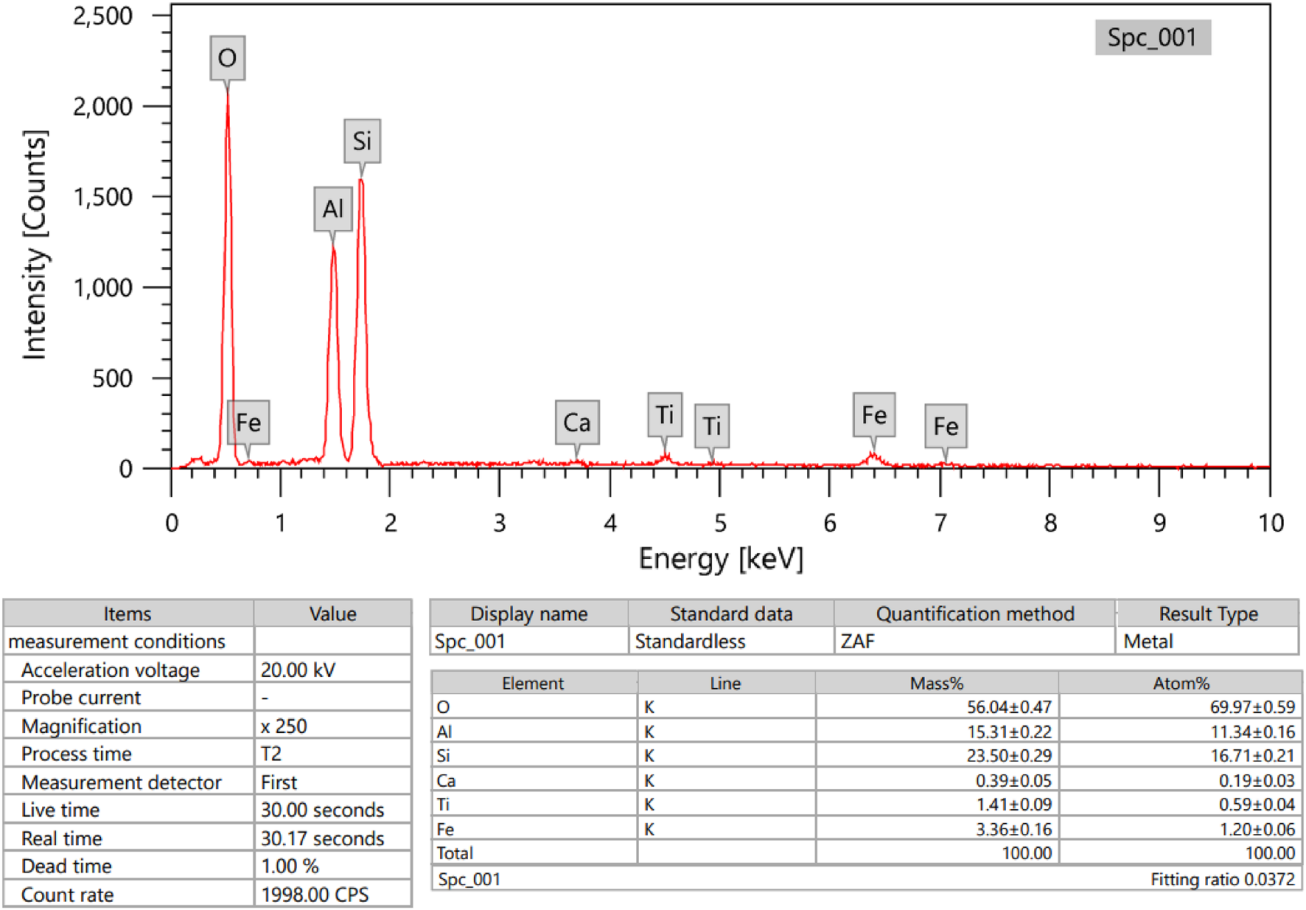
Chemical composition of ball clay (Edx of barite composition as metal).

**Figure 2:**
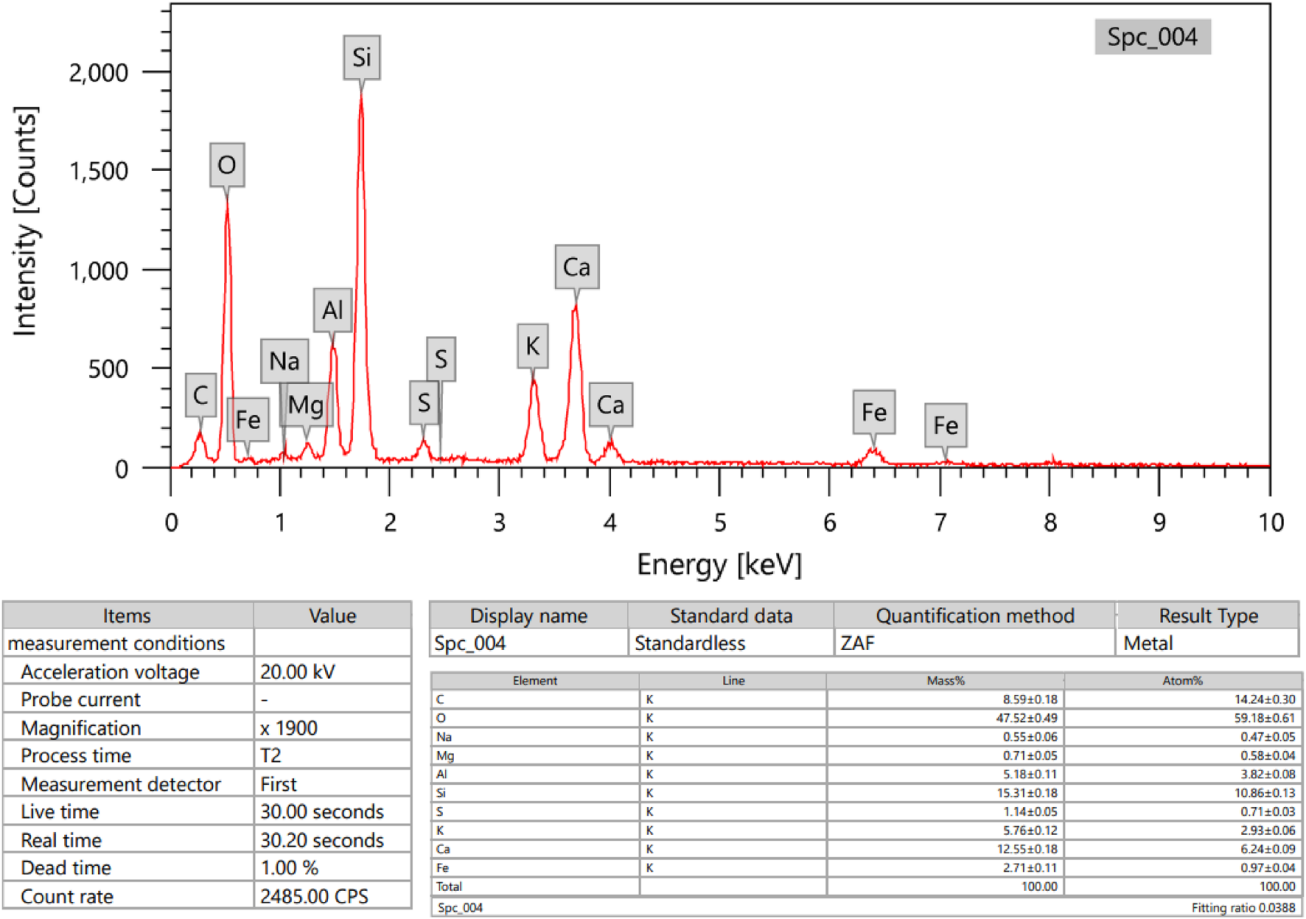
Chemical composition of cement (Edx of barite composition as metal).

In this study, composites are formed from three component: ball clay, cement, and cadmium oxides. First, the ball clay samples were collected from Aswan, Egypt, then dried, crushed, and sieved using a sieve with a hole diameter of 100 µm. In order to ascertain the chemical composition of the ball clay and cement, an Energy Dispersive X-ray (EDX) analysis was used. The findings, which include the average mass percentage of each metal, are available in Figs. 1 and 2.

Secondly, micro-scale metal oxide (CdO) was purchased from the El-Gomhouria Company in Egypt, and its purity was up to 99%. Meanwhile, nano-scale cadmium oxide (CdO) particles were purchased from the Nanotech Company in Egypt, where they were chemically prepared.

### Preparation of cement/ball clay/CdO composites

**Figure 3:**
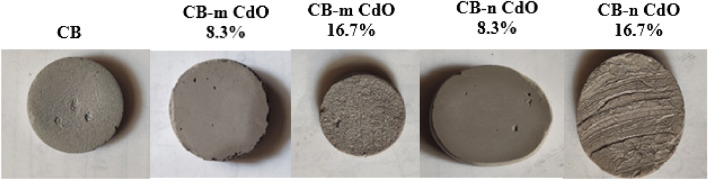
The prepared samples.

The specimens were created using a combination of ball clay, cement, and CdO. The corresponding specimen codes and weight percentages (wt%) of cement, ball clay, and CdO are presented in Table 1. This mixture of powders was weighted sensitively by an electrical balance (Analytical Balance, Japan) with an accuracy of 0.0001 g as it will be added to a proportion of water (mixture:water = 3:1) to form the compound. To shape the specimens, a coin-shaped mold measuring 3 cm in diameter and 0.5 cm in thickness was employed. The mixture was carefully poured into the mold and left to dry and solidify naturally in the open air. Thus, 5 samples were prepared, as shown in Fig. 3.

**Table 1:** Specimen codes and weight fraction in percentage (wt%) of cement, ball clay, and CdO.

Sample codes	Compositions (wt%)		
	Main matrix		Cadmium oxide (CdO)
	Cement	Ball clay	
CS	50	50	0
CB-m CdO 8.3%	50	41.7	8.3
CB-n CdO 8.3%	50	41.7	8.3
CB-m CdO 16.7%	50	33.3	16.7
CB-n CdO 16.7%	50	33.3	16.7

### Instrumentation

#### Gamma ray spectroscopy setup

A high resolution NaI (Tl) crystal, a photomultiplier tube, an aluminum enclosure, and a 14-pin connection are all parts of the Canberra U.S.A. Sodium Iodide Scintillation Detector, model number 802, which is hermetically sealed^21^. The NaI (Tl) detectors of the 802 series offer great efficiency and consistent response in both well and cylindrical formats. These detectors have a track record of stability and long-term dependability^22^. Any Model 802 assembly may be powered by the Model 2007 Tube Base, which plugs into any Model 802 assembly. The Model 802 can also be plugged into the Model 2007P tube base/preamplifier combo. In our research, it was employed a NaI (Tl) detector (802-3 × 3 in) with a resolution of 7.5% at the peak of Cs-137 at 661.66 keV^23^.

**Figure 4:**
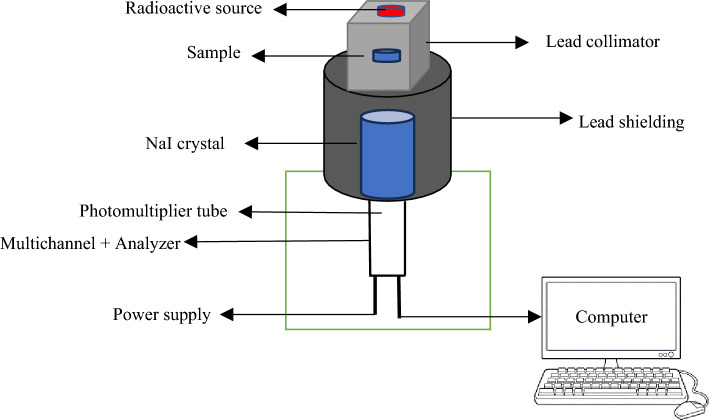
Experimental adjustment for gamma-ray measurement.

Gamma-radiation measurements were made using five standard radioactive point sources in the energy range of 59.53–1408.01 keV. (Am-241, Ba-133, Cs-137, Co-60, and Eu-152). These sources' first activity was 259 kBq, 275.3 kBq, 385 kBq, 212.1 kBq, and 290 kBq. These radioactive sources are now emitting 254.06 kBq, 125 kBq, 292.12 kBq, 43.78 kBq, and 156.85 kBq, respectively^24,25^. The gamma spectra for all the measurements were collected enough times, according to the sample thickness, so that the statistical error would be less than 1%. Genie 2000 program was used to analyze the obtained spectra. The net area under each peak in the spectrum at given energy and thickness were tabulated in an excel sheet to calculate the shielding parameters of the prepared composites. The total standard error has been determined by combining errors for area under peak at given energy, density measurement and thickness measurement. The experimental arrangement for the gamma measurement system is displayed in Fig. 4^26^.

#### Theoretical background

The count rate was calculated in the presence and absence of the sample by Eq. (1)

1 $$\mathrm{I}=\frac{\mathrm{A}}{\mathrm{t}}$$

where A represents area under the curve and t is defined as time to reach error less than or equal 1%^27^.

The $\upmu$ is the linear attenuation coefficient or LAC (${\mathrm{cm}}^{-1}$) is defined as the probability of photons interacting with matter per unit path length and was calculated experimentally by from the well-known Beer–Lambert’s law^17,28^:

2 $$\upmu =\frac{1}{\mathrm{x}}\mathrm{ln}(\frac{{\mathrm{I}}_{0}}{{\mathrm{I}}_{\mathrm{X}}})$$

where ${\mathrm{I}}_{0}$ and ${\mathrm{I}}_{\mathrm{X}}$ are the incident and transmitted intensities, respectively, passing through a target material of thickness x. The mass attenuation coefficient or MAC can be calculated by dividing the experimental linear attenuation coefficient (μ) of a given sample by its density (ρ) as shown in Eq. (3)^29^, and can be obtained theoretically by using XCOM program^30^.

3 $$\mathrm{MAC }=\frac{\mu}{\rho}$$

Other important shielding parameters as HVL and TVL that represent the thicknesses needed to attenuate 50% and 90% of the initial photon intensity, respectively, and can be evaluated by the following Eqs. (4) and (5) respectively^31^.

4 $$\mathrm{HVL}=\frac{\mathrm{Ln}\left(2\right)}{\upmu }$$

5 $$\mathrm{TVL}=\frac{\mathrm{Ln}\left(10\right)}{\upmu }$$

Moreover, MFP is the mean distance a photon travels in an absorber before it undergoes an absorption or scattering interaction that removes it from the initial beam. It is very important characteristic of shielding material that is calculated by Eq. (6)^32^,

6 $$\mathrm{MFP}=\frac{1}{\upmu }$$

Effective atomic number has also been calculated using Eq. (7)^33^:

7 $${\mathrm{z}}_{\mathrm{eff}}=\frac{\sum_{\mathrm{i}}{\mathrm{f}}_{\mathrm{i}}{\mathrm{A}}_{\mathrm{i}}{\left(\frac{\upmu }{\uprho }\right)}_{\mathrm{i}}}{\sum_{\mathrm{i}}{\frac{{\mathrm{A}}_{\mathrm{i}}}{{\mathrm{z}}_{\mathrm{i}}}\left(\frac{\upmu }{\uprho }\right)}_{\mathrm{i}}}$$

where ${\mathrm{f}}_{\mathrm{i}}$, ${\mathrm{Z}}_{\mathrm{i}}$ and ${\mathrm{A}}_{\mathrm{i}}$, refer to the molar fraction, atomic number, and atomic weight of the ith constituent element in the selected composite, respectively.

When choosing a shielding material, the absorption buildup factor (EABF) must be considered to edit the absorption calculations resulting from buildup of secondary photons resulting from Compton scattering^34^. To determine the EABF for the selected composite, the Geometric-Progression fitting method (GP) was employed, and the computations were determined according to the three following steps. The absorption buildup factor is from the significant parameters for the shielding material that will be calculated by using the geometric progression (GP) fitting method carried out in three steps:

First, calculate the equivalent atomic number of the composite, using the following equation^35^:

8 $${\mathrm{Z}}_{\mathrm{eq}}=\frac{{\mathrm{z}}_{1}\left({\mathrm{logR}}_{2}-\mathrm{logR}\right)+{\mathrm{z}}_{2}(\mathrm{logR}-{\mathrm{logR}}_{1})}{{\mathrm{logR}}_{2}-{\mathrm{logR}}_{1}}$$

where ${\mathrm{R}}_{1}$ and ${\mathrm{R}}_{2}$ are the ${(\upmu }_{\mathrm{comp}}/{\upmu }_{\mathrm{total}})$ ratios corresponding to the elements with atomic numbers ${\mathrm{Z}}_{1}\mathrm{and }{\mathrm{Z}}_{2}$ respectively, and R is the ${(\upmu }_{\mathrm{comp}}/{\upmu }_{\mathrm{total}})$ ratio for the investigated composites at a specific energy, which lies between ratios ${\mathrm{R}}_{1}$ and ${\mathrm{R}}_{2}$.

In the second step, after obtaining ${\mathrm{Z}}_{\mathrm{eq}}$ values for the composites and using the GP fitting buildup factors, coefficients (a, b, c, d, ${\mathrm{x}}_{\mathrm{K}})$ in the energies from 0.015 to 15 MeV were calculated using Eq. (9)^36^:

9 $$\mathrm{C}=\frac{{\mathrm{C}}_{1}\left({\mathrm{logZ}}_{2}-{\mathrm{logZ}}_{\mathrm{eq}}\right)+{\mathrm{C}}_{2}\left({\mathrm{logZ}}_{\mathrm{eq}}-{\mathrm{logZ}}_{1}\right)}{{\mathrm{logZ}}_{2}-{\mathrm{logZ}}_{1}}$$

where ${\mathrm{C}}_{1}$ and ${\mathrm{C}}_{2}$ are GP fitting parameters, taken from ANSI/ANS-6.4.3 standard database^37^, corresponding to the atomic numbers ${\mathrm{Z}}_{1}$ and ${\mathrm{Z}}_{2}$ between which ${\mathrm{Z}}_{\mathrm{eq}}$ of the prepared composite.

Third and final step is to calculate the buildup factor using the obtained GP fitting parameters coefficient as follows in Eq. (10):

10 $$\mathrm{K}\left(\mathrm{E},\mathrm{x}\right)={\mathrm{Cx}}^{\mathrm{a}}+\mathrm{d}\frac{\mathrm{tanh}\left(\frac{\mathrm{x}}{{\mathrm{x}}_{\mathrm{k}}-2}\right)-\mathrm{tanh}\left(-2\right)}{1-\mathrm{tanh}\left(-2\right)}, \mathrm{for}\, \mathrm{x}\le 40\mathrm{ MFP}$$

where E is incident photon energy and X is the penetration depth in terms of MFP. So, the calculated build up factor is represented in Eqs. (11) and (12)^38^.

11 $$\mathrm{B}\left(\mathrm{E},\mathrm{x}\right)=1+\frac{\mathrm{b}-1}{\mathrm{K}-1}\left({\mathrm{K}}^{\mathrm{x}}-1\right),\mathrm{K}\ne 1$$

12 $$\mathrm{B}\left(\mathrm{E},\mathrm{x}\right)=1+\left(\mathrm{b}-1\right)\mathrm{x},\mathrm{K}=1$$

## Result and discussion

### Characterization

#### Transmission (TEM) and scanning (SEM) electron microscope

**Figure 5:**
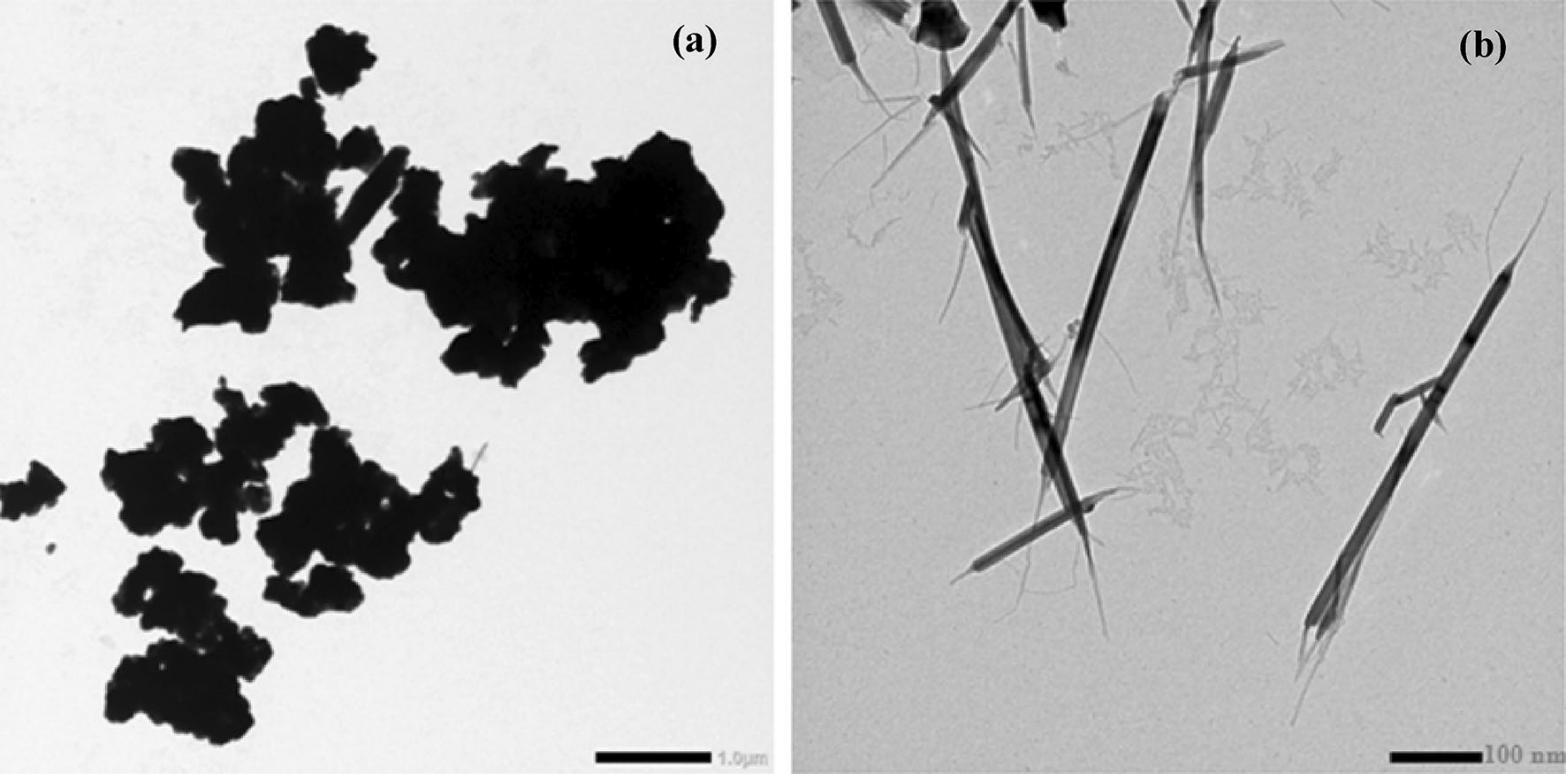
**(a)** SEM image of CdO microparticles, and **(b)** TEM image of CdO nanoparticles.

Figure 5a shows the SEM image of CdO micro particles with a uniform size around 10 µm, while, for TEM micrographs of CdO-nPs, it is noticed that CdO-nPs have a tubed shape with an average particle size around 60 nm, as shown in Fig. 5b.

**Figure 6:**
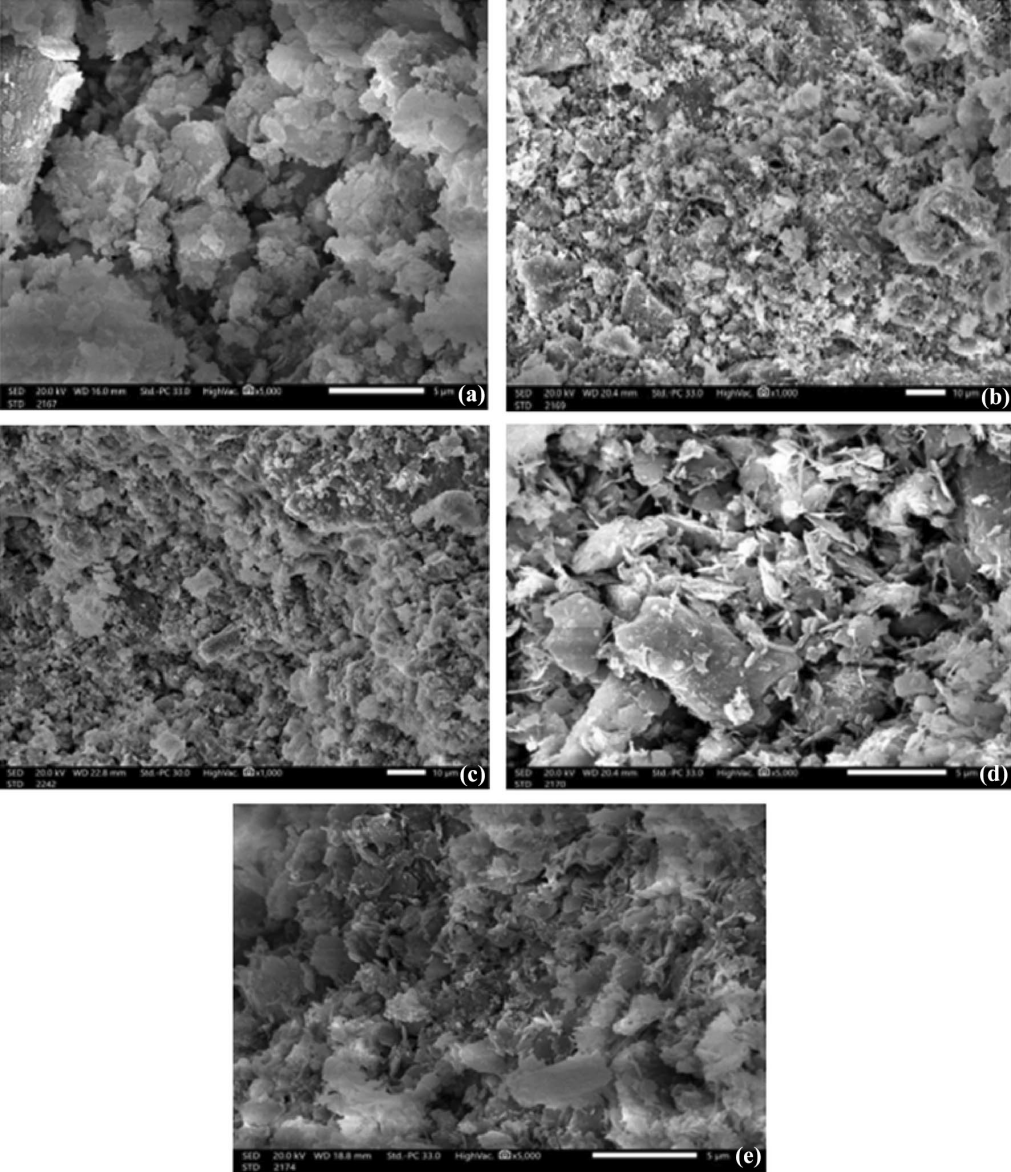
SEM images (**a**) CS (**b**) CB-m CdO 8.3% (**c**) CB-n CdO 8.3% (**d**) CB-m CdO 16.7% (**e**) CB-n CdO 16.7%.

For blank ball clay and cement with CB-m CdO 8.3%, CB-n CdO 8.3%, CB-m CdO 16.7%, and CB-n CdO 16.7%, as shown in Fig. 6 reveals that the clear variation between the morphology of composites without CdO and Nano / Micro composites with different wt.% of CdO.

The CdO NPs are disseminated uniformly in the case of nano-CdO/ball clay composites, which may improve the interfacial adhesion between cement and CdO NPs and offer an interlocking structure for shielding. However, in the case of micro-CdO/ball clay composites, large CdO particles are not well covered by the cement matrix, and some of them peel off from the matrix due to poor interfacial adhesion, acting as voids for shielding. These nanocomposites are anticipated to have superior shielding effectiveness because, as seen in the SEM images, the distribution of nano particles should be more uniform than that of micro particles^39,40^.

#### Mechanical properties

As the composites have different concentrations of micro and nano CdO, it is found that in the low strain region, the composites are uniformly stretched, which follows Hooke’s law. With the increase of micro/nano CdO, the increased content of bound material improves the initial modulus, and with an increase in CdO concentration, the tensile strength and elongation at break are improved. The nanocomposites have a higher tolerance to the applied compression than the micro composites and as the concentration of nano CdO increases, the more brittle the samples become. The ultimate force, stress, and break distance for the composites are shown in Table 2, which shows that the greatest value for the ultimate force was of the CB-n CdO 16.7% sample and the least value was of the CS.

**Table 2:** Ultimate force, stress, and break distance from mechanical compression.

Parameter	Sample				
	CS	CB-m CdO 8.3%	CB-n CdO 8.3%	CB-m CdO 16.7%	CB-n CdO 16.7%
Ultimate force (N)	115.67	712.65	884.12	692.27	1101.00
Ultimate stress (MPa)	2.35	1.45	1.80	1.41	2.24
Break distance (mm)	4.84	3.02	5.56	5.64	2.67

### Gamma ray shielding properties.

The mass attenuation coefficient ${\upmu }_{\mathrm{m}}\left({\mathrm{cm}}^{2}{\mathrm{gm}}^{-1}\right)$, is a widely used parameter in studying and comparing the shielding efficiency of different shielding materials. Table 3 lists out the measured values of mass attenuation coefficients using Eq. (3), theoretical values of mass attenuation coefficients obtained using XCOM program and measured densities of all composites at energies ranges from 59.53 to 1408.01 keV, as well as relative deviation RD (%) that is calculated by the Eq. (14):

**Table 3:** Measured values of MACs, RD%, and density of different concentrations of composites.

Sample	Energy (MeV)	Mass attenuation coefficient (Cm^2^ /gm)		RD%	Density (gm/Cm^3^$$)$$
		Experimental	X-COM		
CS	0.0595	0.3372 ± 0.0023	0.3310	− 1.8880	1.66 ± 0.031
	0.0810	0.2134 ± 0.0001	0.2156	1.0041	
	0.1218	0.1561 ± 0.0001	0.1560	− 0.0466	
	0.2447	0.1137 ± 0.0042	0.1149	1.0782	
	0.3560	0.0976 ± 0.0001	0.0994	1.8069	
	0.6617	0.0776 ± 0.0002	0.0766	− 1.2046	
	0.7789	0.0716 ± 0.0021	0.0712	− 0.5801	
	0.9641	0.0635 ± 0.0004	0.0643	1.3696	
	1.1732	0.0576 ± 0.0001	0.0584	1.3786	
	1.3325	0.0556 ± 0.0001	0.0547	− 1.5658	
	1.4080	0.0535 ± 0.0082	0.0532	− 0.5558	
CB-m CdO 8.3%	0.0595	0.7484 ± 0.0011	0.7390	− 1.2743	1.75 ± 0.04
	0.0810	0.3698 ± 0.0003	0.3853	4.0330	
	0.1218	0.2106 ± 0.0001	0.2073	− 1.5890	
	0.2447	0.1176 ± 0.0001	0.1208	2.6754	
	0.3560	0.0975 ± 0.0033	0.1008	3.2728	
	0.6617	0.0751 ± 0.0011	0.0764	1.7355	
	0.7789	0.0709 ± 0.0015	0.0708	− 0.1111	
	0.9641	0.0614 ± 0.0011	0.0639	4.0226	
	1.1732	0.0559 ± 0.0071	0.0580	3.5044	
	1.3325	0.0538 ± 0.0002	0.0543	1.0007	
	1.4080	0.0524 ± 0.0001	0.0528	0.7943	
CB-m CdO 16.7%	0.0595	1.1743 ± 0.0002	1.1520	− 1.9322	1.86 ± 0.003
	0.0810	0.5489 ± 0.0022	0.5571	1.4795	
	0.1218	0.2607 ± 0.0022	0.2593	− 0.5441	
	0.2447	0.1229 ± 0.0001	0.1267	2.9973	
	0.3560	0.1004 ± 0.0002	0.1023	1.8372	
	0.6617	0.0750 ± 0.0004	0.0761	1.5558	
	0.7789	0.0701 ± 0.0002	0.0705	0.5701	
	0.9641	0.0603 ± 0.0001	0.0635	5.0095	
	1.1732	0.0548 ± 0.0001	0.0575	4.8282	
	1.3325	0.0522 ± 0.0002	0.0539	3.1546	
	1.4080	0.0516 ± 0.0001	0.0524	1.6775	
CB-n CdO 8.3%	0.0595	0.8401 ± 0.0001		12.25209	1.836 ± 0.017
	0.0810	0.4348 ± 0.0001		13.22836	
	0.1218	0.2318 ± 0.0006		10.11393	
	0.2447	0.1344 ± 0.0011		12.83951	
	0.3560	0.1096 ± 0.0004		9.92900	
	0.6617	0.0808 ± 0.0005		7.73285	
	0.7789	0.0748 ± 0.0001		5.56760	
	0.9641	0.0672 ± 0.0003		4.18796	
	1.1732	0.0606 ± 0.0032		6.76679	
	1.3325	0.0560 ± 0.0001		4.24583	
	1.4080	0.0541 ± 0.0001		3.36333	
CB-n CdO 16.7%	0.0595	1.3558 ± 0.0002		15.4660	2.092 ± 0.011
	0.0810	0.6352 ± 0.0022		15.7480	
	0.1218	0.2951 ± 0.0001		13.2110	
	0.2447	0.1418 ± 0.0082		12.7030	
	0.3560	0.1132 ± 0.0011		12.7390	
	0.6617	0.0819 ± 0.0004		9.2799	
	0.7789	0.0751 ± 0.0004		7.3284	
	0.9641	0.0676 ± 0.0005		8.2239	
	1.1732	0.0611 ± 0.0033		8.0491	
	1.3325	0.0558 ± 0.0062		5.3473	
	1.4080	0.0539 ± 0.0003		4.6289	

14 $$\mathrm{RD}\left(\mathrm{\%}\right)=\frac{{\left(\frac{\upmu }{\uprho }\right)}_{\mathrm{XCOM}}-{\left(\frac{\upmu }{\uprho }\right)}_{\mathrm{EXP}}}{{\left(\frac{\upmu }{\uprho }\right)}_{\mathrm{XCOM}}}*100\mathrm{\%}$$

The $\left(\frac{\upmu }{\uprho }\right)$ values obtained using the XCOM database of photon interaction cross-sections relative to the experimental data with gamma-ray energy in the range of 0.059–1.408 MeV.

**Figure 7:**
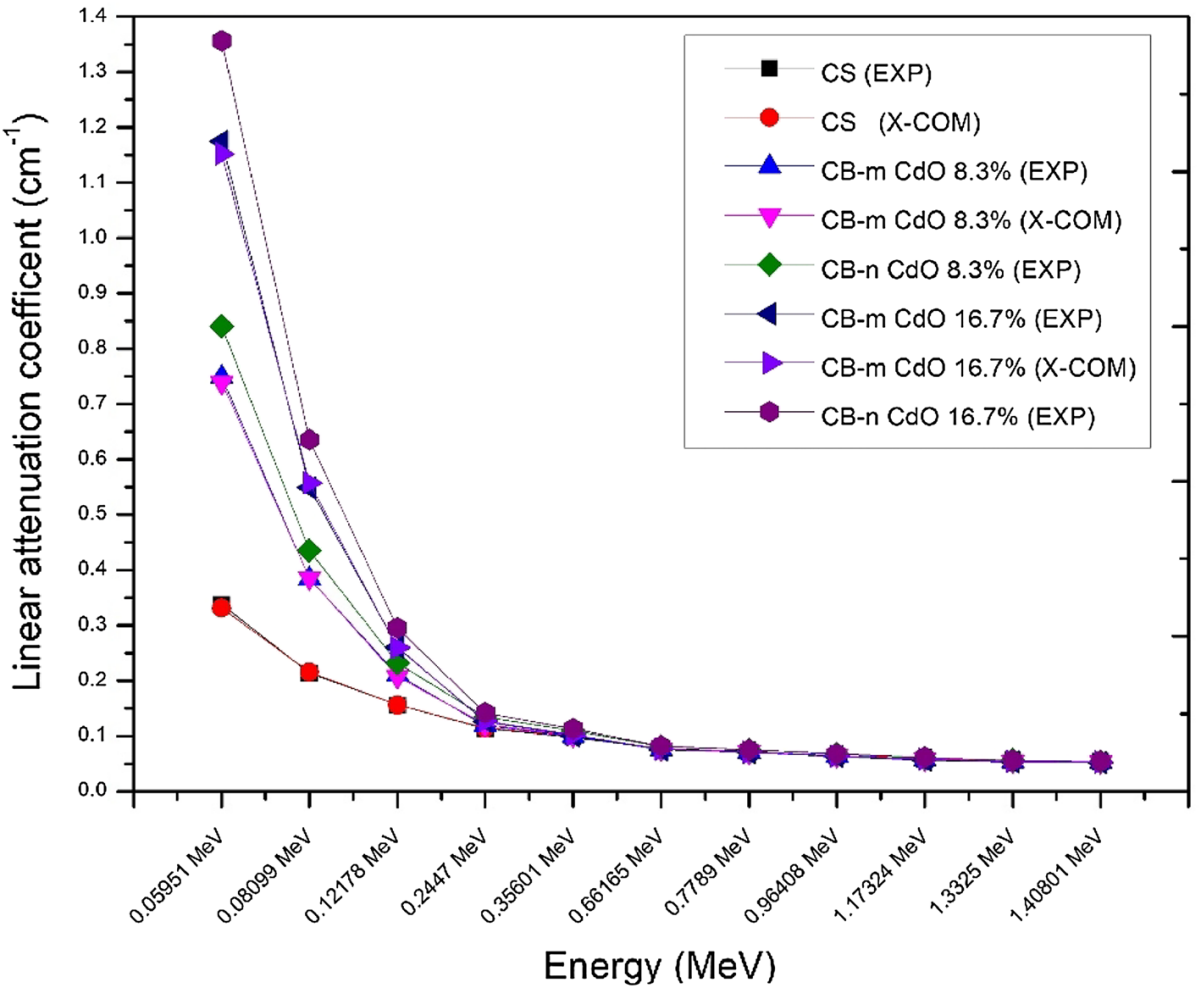
Linear attenuation coefficient of CB-CdO (micro, nano) composites vary with XCOM and experimental.

LAC is represented in Fig. 7 for the energies between 0.059 and 1.408 MeV, which compares the linear attenuation coefficient (LAC) for micro composite against nanocomposites. It can be observed that CB-n CdO has a greater LAC than CB-m CdO for the same weight fraction, and that is due to decreasing the particle size, which increases the surface area per unit volume and consequently increases the probability of interaction. Also, Table 3 demonstrates that the density of cement/ball clay composites increases as the percentage of cadmium oxide in the composites increases. This can be attributed to the high atomic number and density of cadmium oxide. Furthermore, composites filled with nanoparticles exhibit higher density compared to those filled with micro particles at the same weight fraction. Consequently, nanocomposites possess enhanced shielding properties in comparison to micro composites.

**Figure 8:**
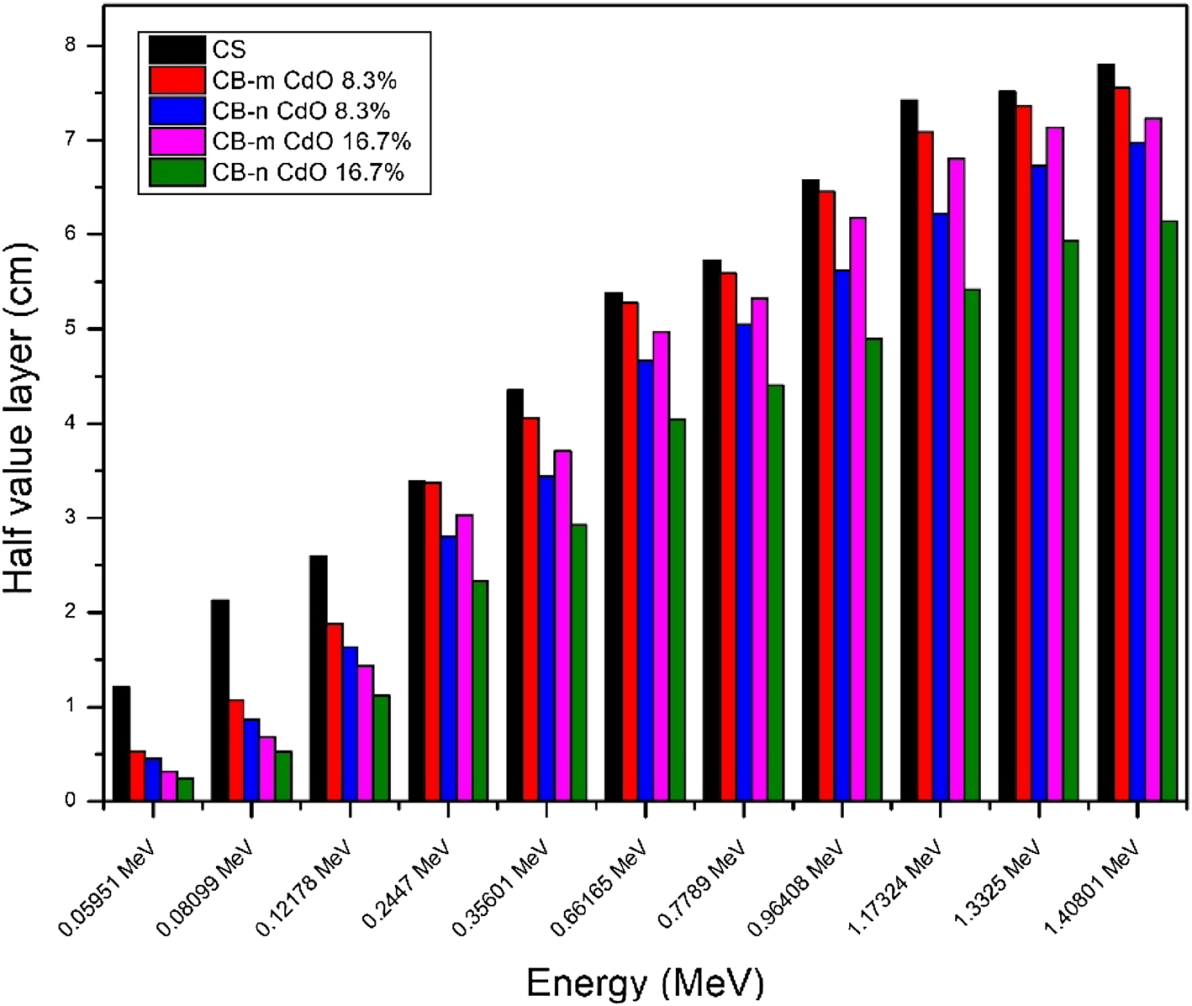
Comparison between HVL for CB-CdO (micro, nano) composites at different energies.

**Figure 9:**
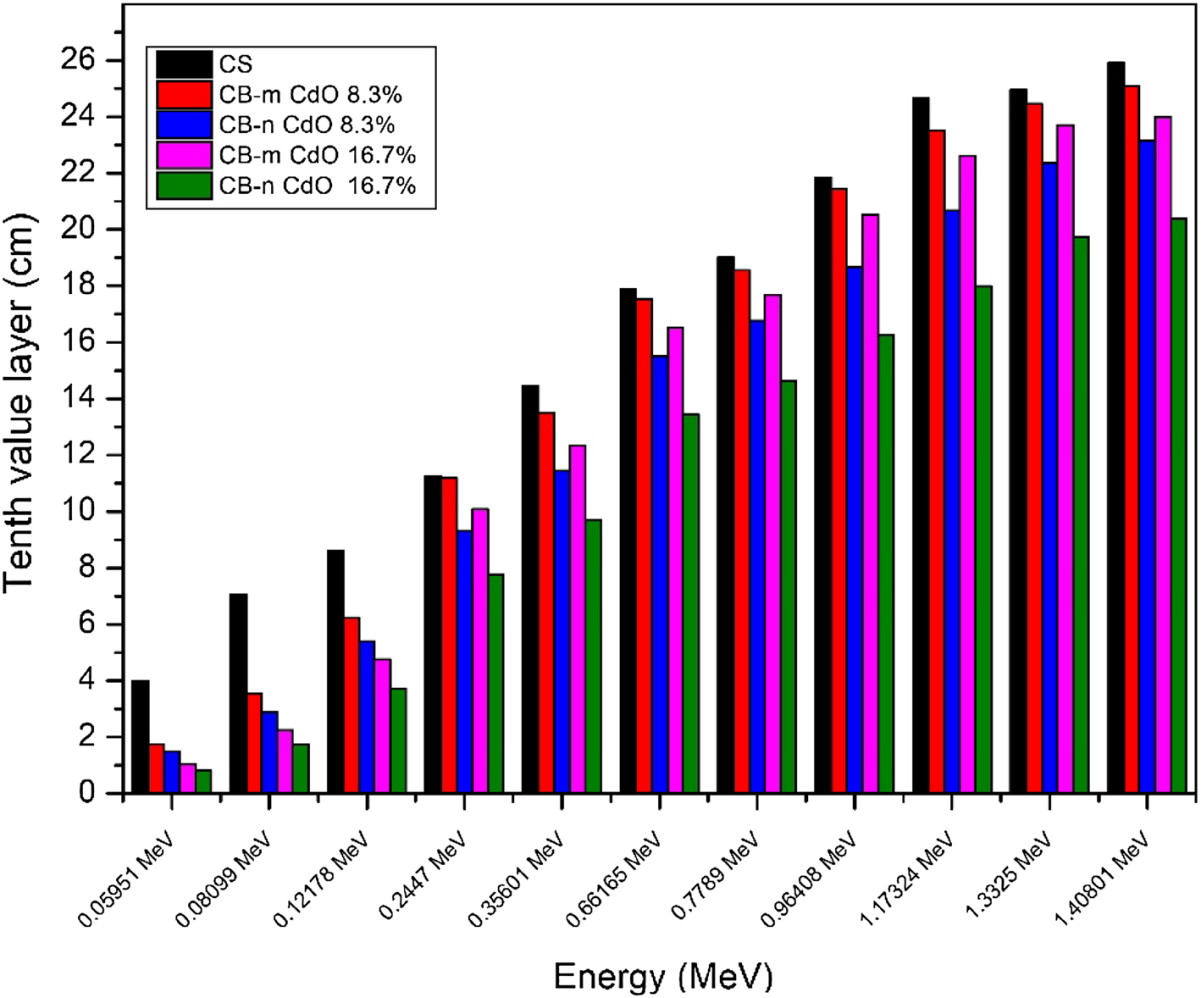
comparison between tenth value layer for CB-CdO (micro, nano) composites at different energies.

**Figure 10:**
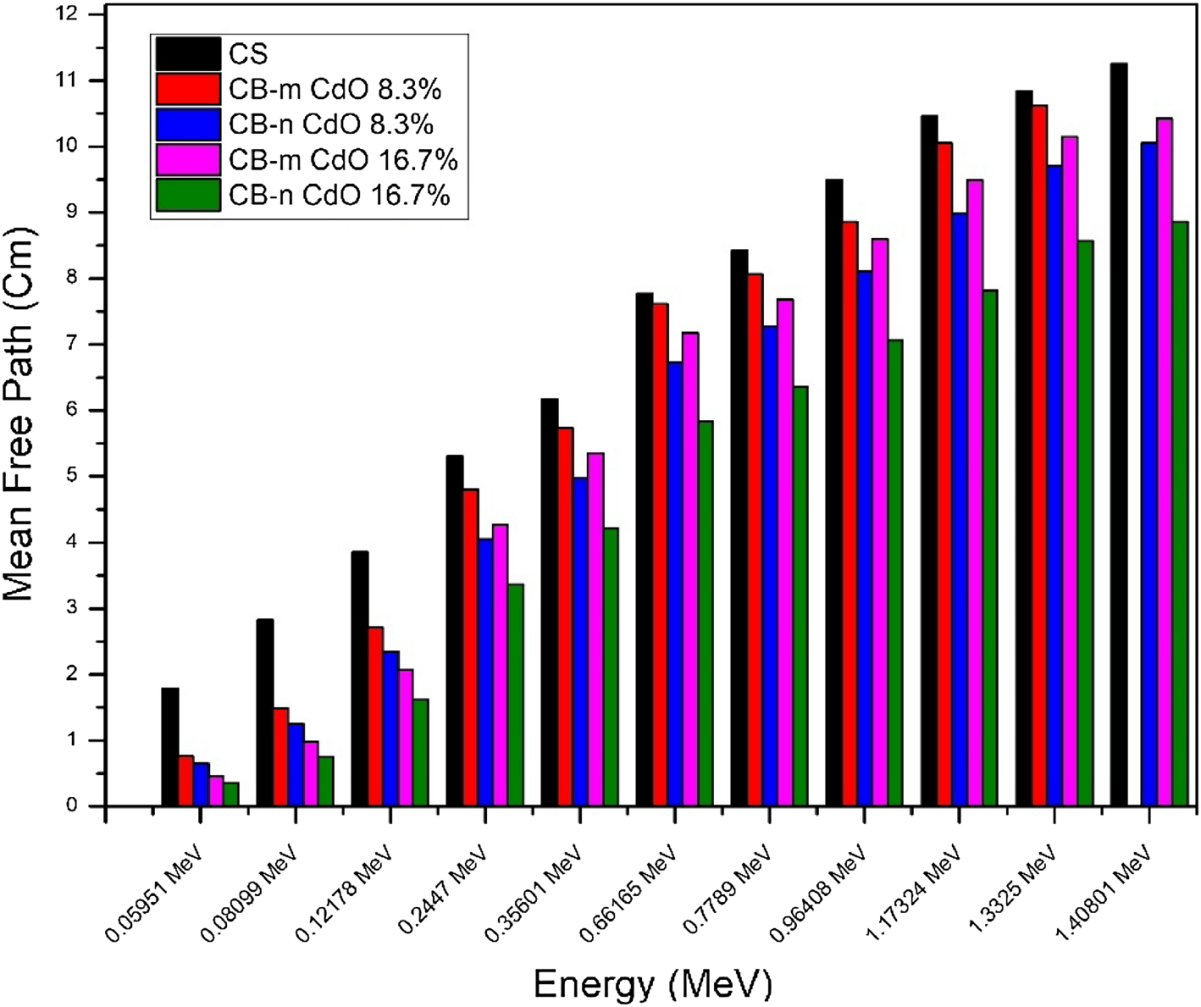
comparison between mean free path for CB-CdO (micro, nano) composites at different energies.

**Figure 11:**
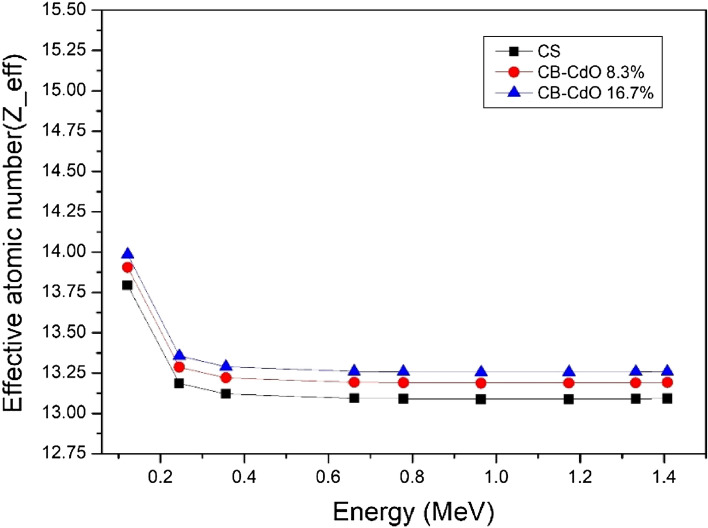
Effective atomic number varies with energy for CB-CdO (micro, nano) composites.

The half-layer value is a very important parameter. Figure 8 shows how HVL for both micro and nano samples has the expected behavior as there is a decrease by incorporating nanomaterials into the shielding material. This reduction in HVL becomes more distinct at high energy than at low energy, showing that by increasing the concentration of nano CdO, the HVL will decrease at the same energy. As well, TVL and MFP are showing the same behavior as shown in Figs. 9 and 10, respectively. The effective atomic number seen in Fig. 11 illustrates the rise in CdO concentration in the CB-CdO composite as the CdO weight fraction increases; this increase is caused by the high atomic number of the Cd element.

**Figure 12:**
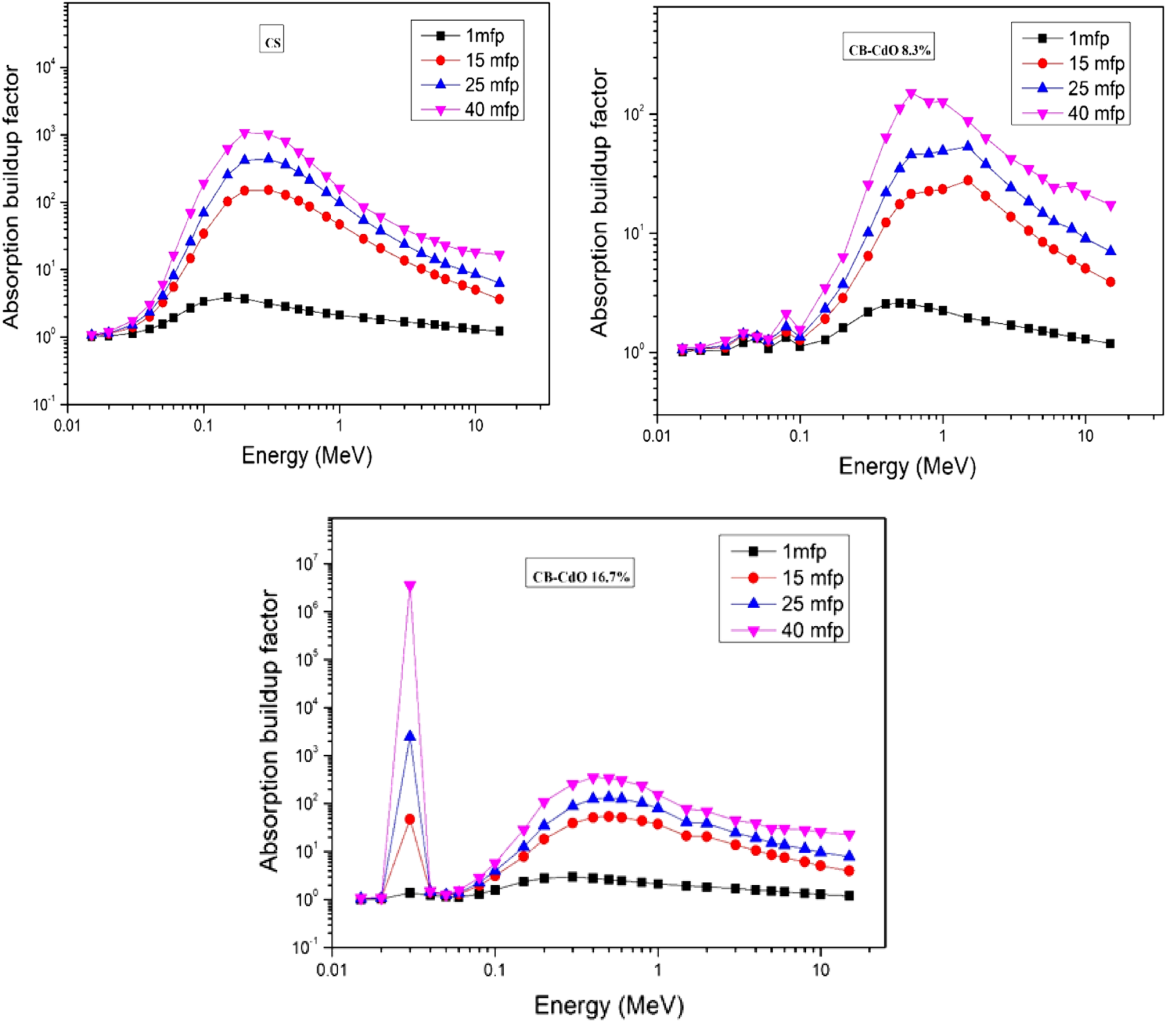
The variation of the EABF with gamma ray energy for cement/ballclay/(micro, nano) CdO composite.

The variation of the buildup factor with a photon energy between 0.015 and 15 MeV is displayed in Fig. 12 at penetration depths 1, 15, 25, and 40 mfp. It is clear from Fig. 12 that the EABF for all composites is small in low energy regions and slightly increases by increasing the penetration depth up to 40 mfp. The photoelectric effects, in which all photons are entirely expelled from the material at low photon energies, could be responsible for these low EABF^41^. In other words, as penetration distance increases, photon interactions with the sample correspondingly increase, causing a significant number of low-energy photons to be generated.

It is also obvious that there is a large peak in the EABF that occurs at 0.21 MeV in all the investigated composites corresponding to K-edge absorption of Cd (0.026 MeV)^42^. This peak is very sharp in CB-CdO 16.7% composites compared with CB-CdO 8.3% composites. Furthermore, the maximum EABF is obtained in the intermediate energy, where the EABF appears to be very large, which is considered a general trend for all the investigated CdO composites. This is due to the Compton scattering process, where the photon is not completely removed but its energy is reduced through multiple scattering, which raises the EABF to a maximum value between 0.21 and 1 MeV. This is attributed to the increase in attenuation probability of photons due to the increase in the concentrations of Cd elements, which is consistent with the former results. Hence, instead of absorbing highly energetic photons (above 5 MeV), the pair production, along with the annihilation process, doubles the photons. In the pair production zone and for deep penetration, it is shown that the EABF values are very high. For the material with the highest equivalent atomic number, an increase in the penetration depth of the material causes a rise in the thickness of the interacting material, which in turn causes an increase in the scattering events in the interacting medium. As a result, the EABF values are high. The EABF curves also resemble those described in the literature for heavy metal oxide in terms of general form.

El-Khatib et al.^17^ used compression-molding technique to prepare pure high-density polyethylene (HDPE), 10 wt%, 20 wt%, 30 wt%, and 40 wt% for micro CdO/HDPE and nano-CdO/HDPE composites. The measured MAC values of the present work were compared with those of (micro and nano) CdO/HDPE composites at different concentrations of wt.10% and wt.20% of (micro and nano) CdO^17^. The relative difference between two different composites is calculated as shown in Table 4. It can be seen that at 0.059 MeV, the MACs of CB-CdO 8.3% (micro and nano) composites are higher than MACs of wt. 10% (micro and nano) CdO with HDPE composites, but for other energies, the MACs of wt. 10% (micro and nano) CdO with HDPE composites are higher than the MACs of (micro and nano) CB-CdO 8.3% composites, but The MACs of wt.20% (micro and nano) CdO with HDPE composites are higher than the MACs of (micro and nano) CdO with CB-CdO 16.7% composites at all energies. But when comparing MACs between (micro and nano) CB-CdO composites at both concentration and particle size with MACs of Cd element from XCOM at different energies. It can be seen that in the low energy, MACs of Cd element are higher than MACs of CB-CdO composites at different concentrations of wt.8.3% and wt.16.7% (micro and nano), but for other energies, MACs of (micro and nano) CB-CdO of wt. 8.3% and wt. 16.7% (micro and nano) CdO are slightly higher than with MACs of Cd element.

**Table 4:** MACs between (micro-nano) CB-CdO composites with measured MACs of (micro-nano) CdO / HDPE composites at different concentration with different energies^17^.

Energy (MeV)	Mass attenuation coefficient (Cm^2^ /gm)								
	10%wt Micro CdO/HDPE^17^	CB-m CdO 8.3%	20%wt Micro CdO/HDPE^17^	CB-m CdO 16.7%	10%wt Nano CdO/HDPE ^17^	CB-n CdO 8.3%	20%wt Nano CdO/HDPE ^17^	CB-n CdO 16.7%	Cd (element)
0.0595	0.691	0.748	1.187	1.174	0.798	0.840	1.374	1.355	5.880
0.0810	0.382	0.369	0.592	0.548	0.439	0.434	0.682	0.635	2.530
0.1218	0.221	0.210	0.282	0.260	0.253	0.231	0.323	0.295	0.856
0.2447	0.135	0.117	0.141	0.122	0.153	0.134	0.160	0.141	0.194
0.3560	0.113	0.097	0.114	0.100	0.128	0.109	0.129	0.113	0.119
0.6617	0.086	0.075	0.085	0.074	0.096	0.080	0.095	0.081	0.073
0.7789	0.080	0.070	0.079	0.070	0.089	0.074	0.088	0.075	0.066
0.9641	0.072	0.061	0.070	0.060	0.079	0.067	0.078	0.067	0.058
1.1732	0.066	0.055	0.064	0.054	0.072	0.061	0.070	0.061	0.052
1.3325	0.061	0.053	0.060	0.052	0.798	0.056	1.374	0.055	0.049
1.4080	0.059	0.052	0.059	0.051	0.439	0.054	0.682	0.053	0.048

## Conclusion

The outcome of this work is that after making the CB-CdO composites with varying concentrations, novel clay materials with enhanced gamma-radiation-shielding features can be fabricated. It was revealed that as the concentration of the substance increased in CdO to 8.3% and 16.7%, the micro and nano samples became brittle. There is a good agreement between the experimental values of MAC for micro composites and those obtained theoretically from the XCOM database. The experimental results demonstrated that the size and concentration of the CdO particles affected the gamma shielding ability of ball clay and cement at all investigated energies. Using SEM, the distribution of nano CdO is more homogenous than that of samples of micro CdO, where nano particles have a high electron density, which results in a higher interaction probability between photons and nano CdO, which improves its ability to shield. Furthermore, it has been observed that the MAC of the nano-CdO composites is significantly higher than that of the micro-sized particles in all samples. Additionally, it is worth noting that the relative deviation percentage of the MAC is also greater for the nano-CdO composites. Moreover, as the size of the CdO particles decreases, the density of the composite material increases. Therefore, it may be inferred that employing nanoparticles rather than microparticles is one way to enhance the shielding probability of materials. Moreover, the HVL, TVL, and MFP values when using nanocomposites were also lower than the values for micro composites, which indicates that nanocomposites attenuated gamma-rays more effectively.

## Data Availability

All data generated or analyzed during this study are included in this published article.
